# Successful staged tricuspid valve replacement following cardiac resynchronization therapy in a congenitally corrected transposition of the great arteries

**DOI:** 10.1002/ccr3.2272

**Published:** 2019-06-24

**Authors:** Seiji Asagai, Daiji Takeuchi, Hisashi Sugiyama, Mitsugi Nagashima

**Affiliations:** ^1^ Department of Pediatric Cardiology and Adult Congenital Cardiology Tokyo Women’s Medical University Tokyo Japan; ^2^ Department of Cardiovascular Surgery Tokyo Women’s Medical University Tokyo Japan; ^3^ Department of Thoracic and Cardiovascular Surgery Wakayama Medical University Wakayama Japan

**Keywords:** cardiac resynchronization therapy, congenitally corrected transposition of the great arteries, systemic ventricular dysfunction, tricuspid valve replacement

## Abstract

Simple tricuspid valve surgery for complex heart disease with systemic right ventricular dysfunction is a high‐risk procedure; however, staged tricuspid valve surgery following cardiac resynchronization therapy seems to be a beneficial method to expect reverse systemic ventricular remodeling.

## 1 INTRODUCTION

Patients with isolated congenitally corrected transposition of the great arteries (ccTGA) are usually asymptomatic in childhood and can survive well into adolescence. Nevertheless, gradual advanced tricuspid regurgitation (TR) and systemic right ventricular (sRV) dysfunction remain the main contributors to mortality and disability in patients with ccTGA after adulthood.^1^, ^2^ Tricuspid valve surgery including tricuspid valve replacement (TVR) is an effective operation for ccTGA with well‐preserved sRV function and TR However, in patients with sRV dysfunction, simple TVR may result in high morbidity and mortality and may not improve pre‐existing sRV failure.^1^, ^2^ Although staged TVR after cardiac resynchronization therapy (CRT) seems to be an alternative method for ccTGA with sRV dysfunction and TR, limited data are available on the treatment results.

## 2 CASE REPORT

**Figure 1:**
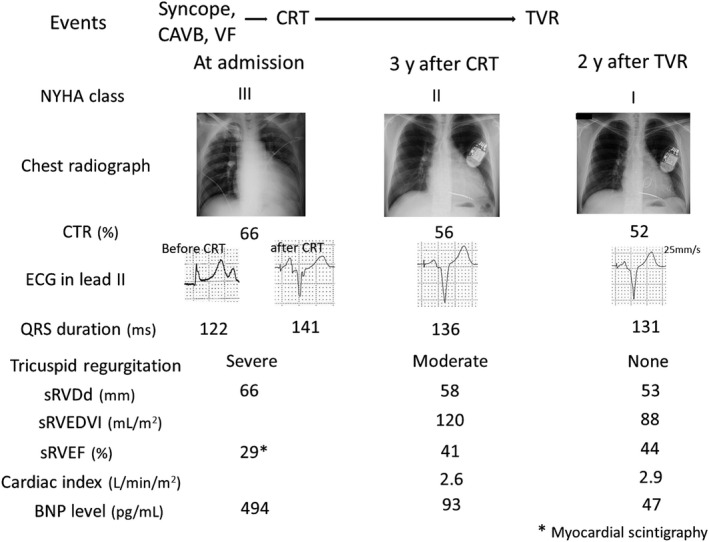
Clinical course before and after tricuspid valve replacement after cardiac resynchronization therapy. BNP, plasma brain natriuretic peptide; CAVB, complete atrioventricular block; CRT, cardiac resynchronization therapy; CTR, cardiacthoracic ratio; NYHA, New York Heart Association; sRVDd, systemic right ventricular diastolic dimension; sRVEDVI, systemic right ventricular end‐diastolic volume index; sRVEF, systemic right ventricular ejection fraction; TVR, tricuspid valve replacement; VT, ventricular tachycardia

**Figure 2:**
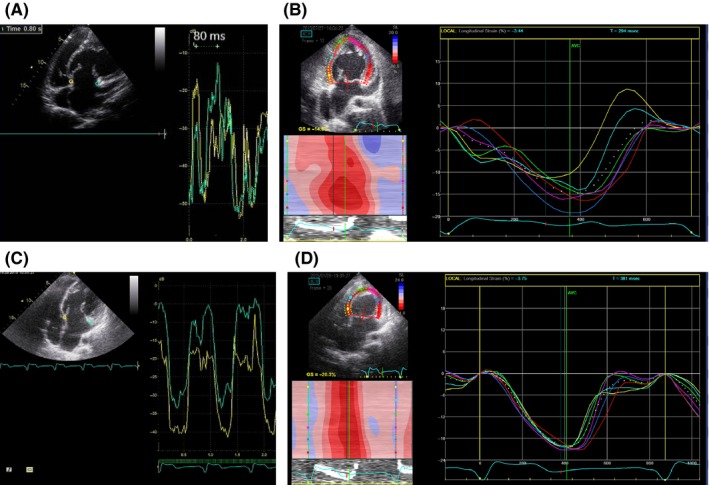
Tissue Doppler imaging and 2‐dimensional (2D) speckle tracking echocardiography before and after cardiac resynchronization therapy (CRT). A, Pre‐CRT tissue Doppler imaging. An intraventricular delay of 80 ms was observed between the end of the right ventricular (RV) free wall contraction and the end of the septal wall contraction on tissue Doppler imaging. B, 2D speckle tracking echocardiography under temporary subpulmonary ventricular pacing. 2D speckle tracking echocardiography showed intraventricular dyssynchrony in the systemic right ventricle. C, Post‐CRT tissue Doppler imaging. After CRT, the intraventricular delay between the end of the RV free wall contraction and the septal wall contraction was improved on tissue Doppler imaging. D, Post‐CRT 2D speckle tracking echocardiography. After CRT, 2D speckle tracking echocardiography showed the disappearance of intraventricular dyssynchrony in the systemic right ventricle

The patient was a 31‐year‐old man with ccTGA, pulmonary artery stenosis, and TR associated with an Ebsteinoid valve diagnosed at the age of 1 month, who had not been followed up with since he was 23 years old. He was referred to our hospital because of syncope. His physical function was classified as New York Heart Association (NYHA) functional class III. Radiography revealed cardiomegaly (cardiacthoracic ratio [CTR], 67%) with pulmonary congestion. Electrocardiography revealed a complete atrioventricular block (CAVB) with a heart rate of 46 bpm and QRS duration of 122 ms and an unstable ventricular tachycardia (VT). His plasma brain natriuretic peptide (BNP) level was 494 pg/mL. Echocardiography revealed a dilated sRV end‐diastolic dimension (sRVDd) of 66 mm, diffuse hypokinesis, and severe TR (Figure 1). An intraventricular delay of 80 ms was observed between the end of the sRV free wall contraction and the end of the ventricular septal wall contraction on tissue Doppler imaging (Figure 2A). He underwent cardiac resuscitation for unstable VT. Therefore, we performed emergency temporary subpulmonary ventricular pacing and subsequently administered amiodarone infusion. Although his bradycardia‐related symptoms and VT were well controlled after temporary pacing with amiodarone therapy, a 2D speckle tracking echocardiography revealed sRV dyssynchrony during single‐site subpulmonary ventricular pacing (Figure 2B).Therefore, we performed a CRT‐D implantation through a transvenous approach to recover sRV function and improve myocardial electrical instability. Before the CRT‐D implantation, we performed a cardiac computed tomography (CT) for the examination of the coronary sinus ostium and major right coronary venous drainage because the incidence of abnormal coronary venous anatomy in ccTGA is high. We confirmed the presence of the coronary sinus ostium in the inferior right atrial septum and proximal right coronary vein drainage to the coronary sinus in this case. After atrial and anatomical left ventricular shock leads were placed on the right atrial appendage and ventricular apex, we performed a coronary vein angiography before coronary sinus lead implantation after coronary ostial cannulation, which confirmed the presence of a lateral branch of the right coronary vein and suggested that a coronary sinus lead could be inserted. We placed a quadripolar coronary sinus lead (1458Q; St. Jude Medical) in the lateral branch, which showed a good pacing threshold without phrenic nerve capture. The Unify Quadra CRT‐D device (St. Jude Medical) was programmed in DDD mode (rate, 70‐140 bpm) with an atrioventricular delay of 110 ms and interventricular delay of −20 ms CRT showed a wider QRS duration (141 ms) than the QRS duration before CRT (122 ms) but a significantly narrower QRS duration than that in single‐site subpulmonary ventricular pacing (170 ms) in lead II. Myocardial scintigraphy with thallium 201 after CRT‐D implantation revealed a low sRV ejection fraction (sRVEF, 29%) and decreased myocardial perfusion from a posteroseptal to an inferoposterior lesion. After CRT‐D implantation, we could safely start pharmacological therapy using a beta‐blocker, angiotensin‐converting enzyme (ACE) inhibitor, diuretics, and amiodarone. Although the QRS duration after CRT was wider than before CRT, both tissue Doppler imaging and a 2D speckle tracking echocardiography revealed an improved intracardiac dyssynchrony (Figure 2C and 2) and an interventricular synchrony (27 ms; normal range, <40 ms) between the systemic right ventricle and the subpulmonary ventricle.

**Figure 3:**
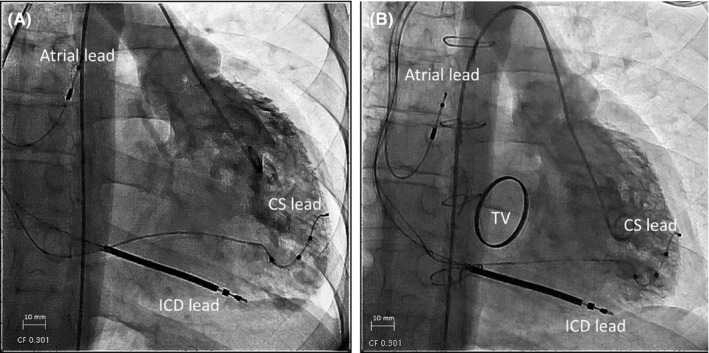
Ventriculography images at the end‐diastolic phase showing a second reverse systemic ventricular remodeling after tricuspid valve replacement. A, Ventriculography image before tricuspid valve replacement (frontal view). B, Ventriculography image 2 years after tricuspid valve replacement (frontal view). CS, coronary sinus; ICD, implantable cardioverter‐defibrillator; TV, mechanical tricuspid valve

At 3 years after CRT‐D implantation, the patient's NYHA classification was class II. The CTR on the chest radiography and the sRVDd on the echocardiography decreased from 66% to 56% and from 66 to 58 mm, respectively (Figure 1). The decreased CTR and sRVDd, despite the persistence of moderate TR, suggested that reverse sRV remodeling occurred as a result of CRT and pharmacological therapy, and we performed cardiac catheterization to determine whether TVR could be performed in this patient. Cardiac catheterization showed a central venous pressure of 4 mm Hg and a cardiac index of 2.6 L/min/m^2^. Although ventriculography revealed a recovery of sRVEF from 28% to 41%, moderate TR and enlarged sRV end‐diastolic volume index (sRVEDVI, 120 mL/m^2^) were still observed (Figure 3). Since the patient showed a good response to CRT and recovered his sRV function, we decided to perform TVR to reduce the sRV volume overload due to TR when the patient was 34 years old. Briefly, after a median thoracotomy and right lateral incision of the left atrium, the tricuspid valve showed hypertrophic leaflets with plastering of the septal leaflet over 10 mm, and the mechanical valve (SJM Masters 31 mm, St. Jude Medical) was placed after removing the septal and anterior tricuspid valve leaflets. During 2 years of post‐TVR follow‐up, no cardiac events occurred, and the NYHA classification improved from class II to class I. At 2 years after TVR, a chest radiography and echocardiography revealed a further decrease in the CTR from 56% to 52% and a decrease in sRVDd from 58 to 53 mm without TR (Figure 1). Cardiac catheterization led to additional increases in the cardiac index and sRVEF from 2.6 to 2.9 L/min/m^2^ and 41%‐44%, respectively, and a decrease in sRVEDVI from 120 to 88 mL/m^2^ (Figure 2). The plasma BNP level was 47 pg/mL.

## 3 DISCUSSION

Tricuspid regurgitation, CAVB, and sRV failure are major complications in adult patients with ccTGA.^3^, ^4^ Previous studies suggested that replacement of the mechanical tricuspid valve in the early stages of sRV dysfunction is superior to performing the replacement in the more advanced stages of sRV dysfunction, because the delayed replacement is associated with a poor prognosis and difficulties in recovering from pre‐existing sRV dysfunction after TVR.^1^, ^2^ In the clinical setting, the combination of advanced TR and sRV dysfunction is a frequent finding in patients with ccTGA. In these cases, cardiac transplantation may be an option for surgical treatment. Cardiac transplantation is an excellent strategy in terms of functional improvement. However, donor organs are insufficient, and cardiac transplantation can only be applied in a limited number of patients.^3^ This is a successful case of a staged TVR strategy after transvenous CRT‐D implantation in a patient with ccTGA with sRV failure, TR, CAVB, and lethal VT. The patient required pacing and medication for CAVB and lethal VT. First, we recommended early CRT‐D implantation because long‐term, single‐site pacing of the subpulmonary ventricle will exacerbate sRV failure.^3^ High prevalence of coronary venous anomalies, such as separate CS ostia, dual CS ostia, and a vein of Marshall without CS ostia in ccTGA, was reported 3, 5, 6; therefore, a review of coronary venous drainage using the levophase of a coronary angiography or CT scan before CS cannulation and coronary venography after CS cannulation is important. His‐bundle pacing is an alternative treatment, especially for difficult cases of CS cannulation owing to an abnormal coronary vein and CS ostium in ccTGA.^7^ In ccTGA, atrioventricular conduction is usually anteriorly deviated; therefore, selective His‐bundle pacing using an ICD lead or even a pacing lead instead of a CS lead seems to be technically difficult and still challenging.^7^ Because this case showed a usual CS ostium on the CT scan, we chose conventional CRT for this case.

We could safely administer the beta‐blocker, amiodarone, and ACE inhibitor owing to reverse tissue and electrical sRV remodeling after CRT‐D implantation. Our case showed reverse sRV remodeling twice: The first included reverse electrical remodeling after CRT‐D implantation,^3^ and the second occurred after TVR owing to reduction in sRV volume overload. A failing systemic right ventricle may achieve less reverse ventricular remodeling after CRT than a systemic left ventricle.^8^ Although the reason for the excellent reverse sRV remodeling in this case is unknown, we presumed that irreversible fibrotic myocardial degeneration still occurred minimally and that electrical remodeling and myocardial ischemia due to increased ventricular wall stress in the hypertrophic sRV wall caused by bradycardia were mainly associated with sRV dysfunction. CRT allowed guideline‐directed medical management and control of bradycardia without inducing intraventricular dyssynchrony by single‐site pacing, even with a wider QRS duration after CRT than before.^9^ Only one‐site pacing at the subpulmonary ventricle exacerbated the sRV function.^10^

## 4 CONCLUSION

Staged TVR after CRT is a valid option for rescuing a ccTGA patient with TR and sRV dysfunction. Further larger studies are required to establish the long‐term efficacy of this strategy in ccTGA.

## CONFLICT OF INTEREST

None declared.

## AUTHOR CONTRIBUTION

SA DT, HS, and MN: conceived and designed the study. SA and DT: acquired, analyzed, and interpreted the data. SA and DT: drafted the manuscript.
